# A perennial living mulch system fosters a more diverse and balanced soil bacterial community

**DOI:** 10.1371/journal.pone.0290608

**Published:** 2023-08-29

**Authors:** Hanxia Li, Nicholas Hill, Jason Wallace

**Affiliations:** 1 Institute of Bioinformatics, The University of Georgia, Athens, GA, United States of America; 2 Crop and Soil Sciences, The University of Georgia, Athens, GA, United States of America; University of Salento: Universita del Salento, ITALY

## Abstract

Cover crops are known to positively impact soil health, both at a physical level (through erosion control and organic matter enhancement) and at a biological level (by fostering more diverse microbial communities). However, most research in this area has been conducted in the context of annual cover crops that are terminated when the main crop is planted. We have previously demonstrated that a continuous “living mulch” cover crop system can enhance the physical and chemical aspects of soil health; In this study, we reveal its effect on the soil bacterial community and compare it to two different annual cover crops and a conventional control without cover crops. We examined the effect of a living-mulch (LM) system using perennial white clover (Trifolium pratense L), annual cereal rye (Secale cereale L.) (CR), annual crimson clover (Trifolium incarnatum L.) (CC), and a no-cover (NC) control at three time points during the 2018 growing season. 16S rRNA amplicon analysis of the soil bacterial community revealed that the community composition in cover crop systems was significantly different from the NC control, and that LM and CR accommodated more heterogeneous and even bacterial communities compared to the NC control. The difference in bacterial composition between cover crop systems appears to be partly influenced by soil nitrogen concentration and lime buffer capacity. Overall community diversity was associated with nitrogen and metal ion concentrations, and these associations were both stronger and more numerous later in the season. These results elucidate how a perennial cover crop system affects the soil bacterial community and advance our understanding of the interactions between crops, management practices, and soil microbiomes in sustainable agriculture.

## 1. Introduction

The world population is projected to reach 9.7 billion in 2050 and 11.2 billion in 2100 [1]. As a result, the need for food is expected to increase by 59–98% between 2005 and 2050 [2]. Until now, global agriculture has managed to sustain the human population by using practices such as high fertilizer input and intensive cropping. However, these practices come at the cost of soil health and productivity, resulting in soil erosion, water pollution, salinization, desertification, and nutrient leaching [3–5].

To address the food demand in the 21st century, agriculture will need to adopt practices that increase crop production while maintaining soil productivity and avoiding environmental damage. Although there are many methods to approach this goal, one of particular interest is the use of cover crops. Cover crops have clear benefits for soil health by preventing soil erosion, reintroducing soil organic matter, and preventing nutrient leaching [6–9]. Although most cover crops are killed before the main crop is planted, it is possible to use them in a “living mulch” system where the target crop (maize, cotton, etc.) is planted into a living, perennial cover crop that is allowed to keep growing alongside the main crop.

We have previously published on a living mulch system based on establishing a solid stand of a perennial legume, white clover (*Trifolium repens* L) [10]. Briefly, this system starts by seeding clover in the fall and allowing it to grow during a mild winter, such as occurs in the southeastern United States. In the spring, strips of clover are killed, and the primary crop (maize in this case) is strip-planted into these gaps. As the primary crop grows, it shades the clover, forcing leaf senescence and nutrient release. After the primary crop is harvested, the remaining clover plants regrow and cover the ground during the mild and moist winter in the southeastern United States. Unlike annual cover crops such as cereal rye and crimson clover, which are often planted in late summer and removed before planting the main crop the next year, this system provides year-round protection from soil erosion and supports corn growth with a significantly lower amount of N fertilizer inputs [10].

Previous work has shown that this system can significantly impact soil health as measured by various chemical and physical characteristics [11]. We were also interested in characterizing its effect on soil microbial communities, which are known to support plant growth [12]. Aspects of the microbial community, such as species richness, diversity, and evenness, have been identified as essential factors in soil health and plant productivity [13]. For example, the initial evenness of the microbial community—meaning how evenly distributed different taxa are—strongly influenced the ecosystem stability under salinity stress [14]. Many studies have looked at the effect of cover crops on the soil microbiome[15–18], and the general trend is that cover crops increase soil microbial diversity, promote microbial nutrient cycling (such as carbon, nitrogen, and phosphorus), and suppress potential pathogens[19, 20]. However, Individual systems can go against these trends, for instance, when one study found that a radish-based cover crop benefitted microbial abundance and richness but reduced evenness among fungi [21]. Another study, meanwhile, found that a perennial cover crop in Chinese pitaya (dragonfruit: *Selenicereus* spp.) orchards increased the presence of potentially beneficial fungi but decreased available phosphorus and potassium [22].

Most cover crop studies focus on annual cover crops, such as cereal rye, annual clover, or brassicas [20]. The study of perennial cover crops seems largely confined to perennial cropping systems, such as trees, though these systems also often use annual covers [21, 23]. The use of perennial cover crops in annual cropping systems, such as we describe here, is much less studied and usually focuses on the agronomic properties of the system (weed control, water infiltration, yield, etc.) An exception was a study that reported that perennial white clover increased maize yield via associations with arbuscular mycorrhizal fungi [24]; however, the researchers did not look at the bacterial community.

To better understand how the perennial living mulch cover crop affects the soil microbiome, we evaluated the long-term effect of living mulch on the soil bacterial community and compared it with traditional cover cropping and no-cover practices after four years of continuous cultivation. As a perennial legume cover crop, living mulch has the potential to provide more organic matter back to the soil and fix more nitrogen than annual legume and non-legume cover crops. Each system in this study follows slightly different management practices (especially regarding nutrient inputs), which reflect how they would actually be used in farmers’ fields. Thus, these experiments are meant to test the entire system, not just an individual component like nitrogen application or cover crop species. We hypothesized that these different agronomic systems would result in different soil chemical and biological properties and that the biological properties would be at least partly influenced by the chemical ones. We further hypothesized that the living mulch system would result in more nutrient availability, a more diverse soil microbiome community, and better soil quality when compared to plots with no cover crop.

## 2. Methods

### 2.1. Field history

The experiment plots were described previously [10, 11]. In brief, before the experiment started, the plot location was planted with pearl millet [*Pennisetum glaucum* (L.) R.Br.] in the summer of 2012, soft red winter wheat (*Triticum aestivum* L.) in the fall of 2012 and spring 2013, grain sorghum [*Sorghum bicolor* (L.) Moench] in the summer of 2013, and cereal rye (*Secale cereale* L.) in the fall of 2013 and spring 2014. The plot was left fallow during the summer of 2014. Plots were divided, and the initial cover crops were planted in the fall of 2014.

### 2.2. Field layout and setup

The field design has been described previously [10, 11]. In brief, plots were laid out in a modified randomized complete block design with three subplots for each cover crop system (S1 Fig in S1 File**)**. Plots were located at the J. Phil Campbell Research and Education Center in Watkinsville, GA (33°52´9.5˝ N 83°26´59.8˝ W; 219 m elevation). Pedological analysis confirmed the soil as a Cecil sandy loam (fine, kaolinitic, thermic typic Kanhapludults). Plots were 6.1 × 7.3 m and designed for eight rows of corn per plot. The experiment started in 2014 and ended in 2018. Initially, it aimed to compare just three cover cropping systems: (1) a perennial white clover (*Trifolium pratense* L.) living mulch (LM) system; (2) an annual cereal rye (*Secale cereale* L.) (CR) system, and (3) an annual crimson clover (*Trifolium incarnatum* L.) (CC) system. Adjacent No Cover (NC) control plots were added in 2016 after the cover crops had been in production for two years. All cover crops were planted on October 17th. 2014. In early the following spring, CC and CR plots received a broadcast herbicides application of glyphosate [N-(phosphonomethyl) glycine] and dicamba (3,6-dichloro-2-methoxybenzoic acid) to suppress cover crops for corn that would be planted in late April. Herbicide was applied to LM plots in 20-cm band centered on 90-cm rows. Both annual cover crops (Wrens Abruzzi CR and Dixie CC) were hand-seeded at rates of 100 and 28 kg ha^–1^, respectively (USDA-NRCS, 2015). The corn variety was DeKalb DKC64-69 with GENVT3P and was planted in late April each year. The second herbicide application of pendimethalin (3,4-dimethyl-2,6-dinitro-N-pentan-3-yl-aniline) and atrazine (1-chloro-3-ethylamino-5-isopropylamino-2,4,6-triazine) was applied to all plots at the vegetative emergence stage of maize. Herbicides were applied in a broadcast manner in CC and CR systems and limited to a 20-cm cow band in the LM plots. Annual cover crops were reestablished every year in the same plot areas and at the same seed rates. LM can regrow in the mild winter of the southeast US and forms a dense canopy in spring without seeding.

Aside from the cover crop, the primary difference among the systems is the amount of nitrogen fertilizer applied, since the legume covers (CC and LM) supply nitrogen through fixation. LM, CC, CR, and NC systems received a total fertilization of 45, 168, 280, and 280 kg N ha^–1^, respectively, using by a dry urea (340 g N kg^–1^) fertilizer approximately 5 cm to one side of the cornrow. The CR and CC plots received 56 kg ha^–1^ N at planting and 224 kg ha^–1^ N at the V6 stage of corn growth. LM and CC plots, respectively, received 45 kg and 168 kg N ha^–1^ at the V6 stage of corn growth. These different nitrogen rates are consistent with actual cropping practices using these systems since the goal was to compare the systems as a whole and not just the cover crop species.

### 2.3. Sample collection

A total of 36 soil samples were collected on May 21st, June 28th, and August 31st of 2018 (12 samples at each time point) after the cover crops had been in place for four years and the no-cover comparison plots for two years. These times correspond to roughly 600 (May) and 1400 (June) growing degree days after planting and also immediately (<24 hours) after corn harvest (August). Each set of 12 samples contained all four systems—NC (control), CC (annual), LM (perennial), and CR (annual)—each with three biological replicates. A jab-style soil sampler was used to collect ten soil cores (6 inches deep, 1 inch in diameter) between the central two rows of each plot. Soil was placed into plastic bags, homogenized, and a subsample was taken to fill a 1.5 ml microtube. To avoid the potential of earlier samples disrupting the soil microbiome for later samples, samples later in the season were carefully collected in different areas from the previous ones. All samples were stored in a cooler filled with dry ice during the collection process, then placed in a lab cryogenic freezer at -80°C until DNA extraction.

Samples for soil physical and chemical analysis were taken separately and have been reported previously [11]. In brief, eight soil cores samples (15 inches deep, 1.5 cm in diameter) for chemical analysis were randomly collected from each plot on May 18th, June 18th, and July 18th in 2018. The combined, pulverized, and dried soil was analyzed for chemical properties (texture, micro- and macro-nutrients, etc.) as a fee-for-service at UGA’s Soil and Plant Testing Lab. Two surface soil cores from each plot were sampled after the final harvest in 2018 to measure water-holding capacity, soil porosity, and saturated surface infiltration rates (Ksat).

### 2.4. DNA extraction and 16S rRNA sequencing

DNA was extracted from thawed soil samples with a Quick-DNA Fecal/Soil Microbe 96 Kit (Zymo), following the manufacturer’s instructions. 16S rRNA gene amplification was performed using the Earth Microbiome Project 515F [25] and 806R primers [26] with Illumina linkers. The exact sequences are included in the S1 Appendix. Peptide nucleic acids (PNA Bio) were mixed and diluted to 2.5uM each for inclusion in the reaction. pPNA (ggctcaaccctggacag) and mPNA (ggcaagtgttcttcgga) were used to block plastid and mitochondrial amplification, respectively. The first PCR reaction consisted of 5 μL DNA template, 2 μL of each primer (0.5 μM), 12.5 μL of Hot Start Taq 2X Master Mix (New England Biolabs), 2.5 μL PNA mixture (2.5 μM each), and 1uL of sterile water. The PCR thermocycle program was 95°C for 45 seconds; twenty cycles of 95°C for 15 seconds, 78°C for 10 seconds, 60°C for 45 seconds, 72°C for 45 seconds; and finally held at 4°C. PCR products were purified with AMPure beads (Beckman Coulter Life Sciences). 5 μL of the first PCR product for each sample was used in the second PCR amplification. The reaction mixture consisted of 5 μL first PCR product, 5 μL Nextera i5 and i7 Barcode Primers, 12.5 μL 2x Taq DNA polymerase master mix, and 2.5 μL PNA mix. The PCR thermocycle program was 95°C for 45 seconds; 25 cycles of 95°C for 15 seconds, 78°C for 10 seconds, 60°C for 45 seconds, 72°C for 45 seconds; and finally, 68°C for 5 minutes followed by holding at 4°C. The second PCR products were purified using AMPure beads, and the cleaned products were eluted in 27uL of sterile water. 25 ul of the solution from each sample, which is the final 16S amplicon sequencing library, was stored in the freezer at -20°C until sequencing. Three blanks were used in DNA extraction and library preparation. Libraries were sequenced at the Georgia Genomics and Bioinformatics Core on an Illumina MiSeq instrument using one paired-end 250 flowcell. Raw sequencing data are deposited in the NCBI SRA database (accession: PRJNA734906).

### 2.5. 16S rRNA analysis and taxonomy classification

16S rRNA classification was performed using the Deblur pipeline [27] in QIIME2 [28]. In brief, Cutadapt was used to remove adapters from raw 16S amplicon reads [29]. The trimmed reads pairs were then joined and imported into QIIME2 software. Reads were filtered based on quality score and trimmed to 240 bp length to avoid low-quality regions near the tail of the reads. Amplicon sequence variants (ASVs) were called using Deblur. ASVs that were present in blank wells were considered contamination and were filtered out for downstream analysis using custom python scripts. Alpha diversity rarefaction analysis was performed in QIIME2 on the filtered ASV tables. One outlier sample (GA18-20S-600 from the May 21^st^ sampling) was excluded from downstream analysis due to extreme differences from all other samples. Taxonomy for each ASV was assigned using a prebuilt 515F/806R region classifier based on the Silva v138 99% ASVs dataset [30] and supplied on the QIIME2 data resources page (https://docs.qiime2.org/2021.2/data-resources/). Finally, any mitochondria and chloroplast sequences were filtered out.

### 2.6. Community analysis

QIIME2 was used to calculate the alpha and beta diversity of the dataset at a rarefied sequence depth of 19,000 reads per sample. This depth was chosen based on the rarefaction curve for these samples (S2 & S3 Figs in S1 File), which indicated that it would capture the majority of diversity in these communities while excluding a minimum number of samples. Differences in community alpha diversity were determined based on linear regression, while differences in beta diversity were determined by pairwise PERMANOVA tests. A basic linear regression model was used to test the effect of cover crop systems, sampling date, and their interaction on alpha diversity. Two samples (GA18-21S-LM1400 and GA18-22S-600) were removed from the linear regression analysis based on strongly violating linear regression assumptions in diagnostic checks (S4 Fig and S1 Table in S1 File), as determined with the gvlma package in R [31, 32]. After removing these samples and confirming the assumptions were satisfied, Type 2 and Type 3 ANOVA were performed using the R package car [33] to determine the contribution of each system to alpha diversity.

### 2.7. Differential abundance analysis and association with soil chemical data

The assigned taxonomy, ASVs, and phylogenetic tree from QIIME2 were imported using the Phyloseq package [34] in R, and DEseq2 was used to determine differentially abundant ASVs between cover-crop systems [35]. Both individual ASVs and family-level groupings were tested at adjusted p ≤ 0.05. The association between each family taxa and soil chemical data was analyzed with a linear mixed model using the Massalin2 package [36] in R. To test the relationship of bacterial families to soil parameters, the following equation was fit for each soil chemical parameter: the abundance of family-level taxa ~ fixed effects (soil chemical parameters) + random effects (replicate + date taken). Due to the large number of tests performed, the resulting p-values were corrected to false discovery rates [37].

### 2.8. Relationship between soil chemical data and diversity

A linear regression model that fit each soil parameter as a function of the cover-crop system, sampling time, and their interaction was used to analyze the contribution of each term on soil parameters. The gvlma package in R was used to confirm no strong violation of linear regression assumptions in each model. The Emmeans packages [38] in R were then used to estimate the marginal mean of each system’s effects on soil chemical data. The correlation between soil chemical data and alpha diversity was examined by both linear regression models in R and by a Spearman correlation in QIIME2, using the alpha correlation function. The resulting p-value was corrected for multiple testing using False Discovery Rate.

To fully evaluate the relationship between soil chemical parameters and microbial communities, we tested the relationship between them with three different methods. The datasets were separated by sampling date to avoid the correlation of time-series samples. First, the beta correlation function in QIIME2 was used to build the distance matrix based on soil chemical data and perform a Mantel test against bacterial beta diversity distance matrices (Bray-Curtis, weighted UniFrac, and unweighted UniFrac distances, Aitchison distance). Second, a basic linear regression model was also used to examine the relationship between the soil chemical data distances and beta diversity distances. Finally, the enfit () function in Vegan [39] was used to associate soil chemical properties with non-metric multidimensional scaling (NMDS) of the bacterial data, which produces an ordination based on the input distance matrix. All bioinformatics scripts for this project are available at https://github.com/wallacelab/paper-li-living-mulch-2021. All R language Scripts were written in R version 3.6.3.

## 3. Results

### 3.1. Cover crops change soil chemical parameters

**Fig 1:**
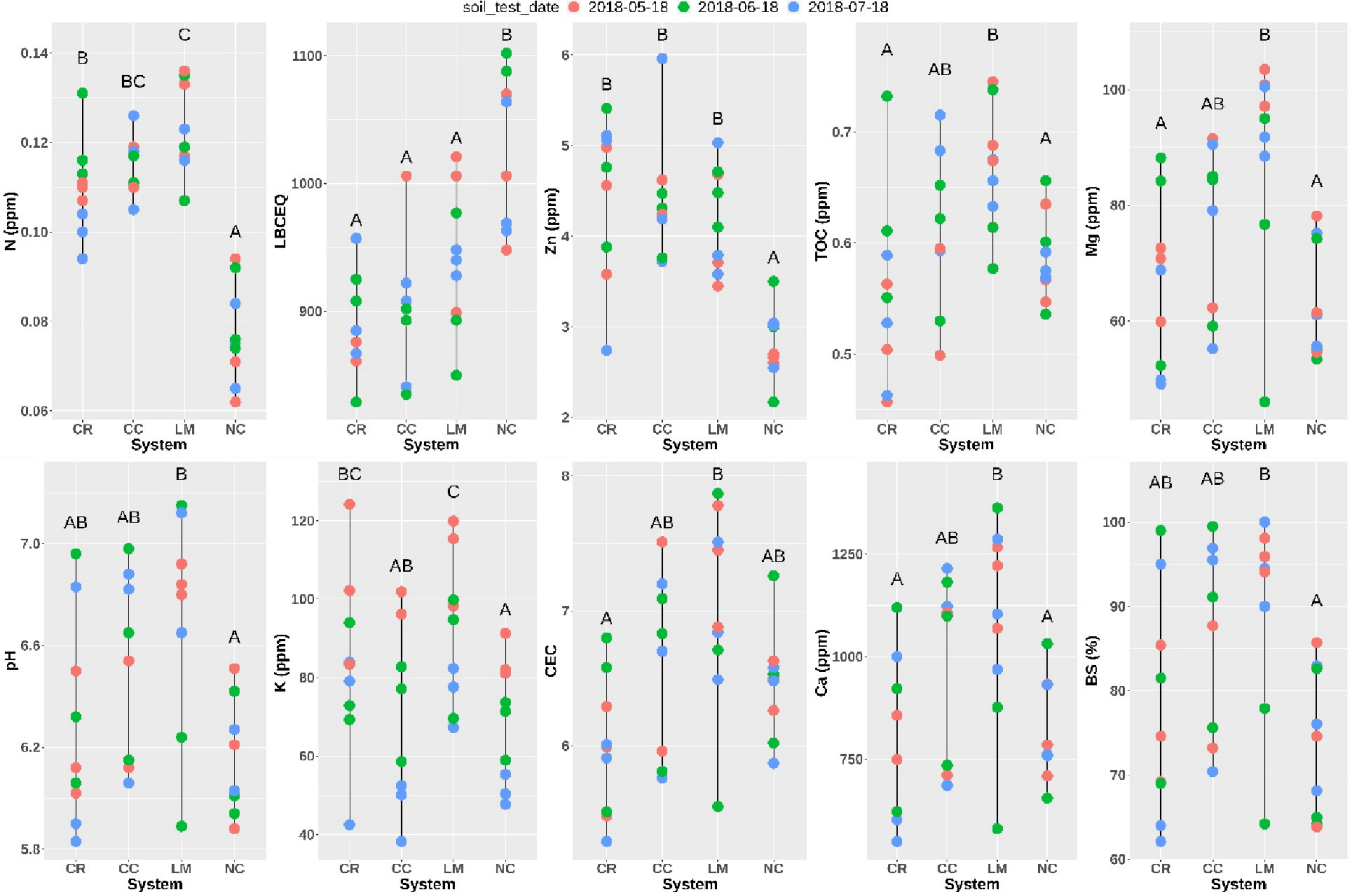
Soil chemical data in 2018. TOC = total organic carbon. LBC = lime buffer activity (pH buffer capacity). CR = CerealRye, CC = CrimsonClover, LM = LivingMulch, NC = NoCover. LBCEQ = lime buffer capacity equilibrium value. No significant difference exists between the systems in soil nitrate, calcium, phosphate, concentration and base saturate percentage.

The soil chemical parameters on May 18th, June 18th, and July 18th in 2018 have been reported previously [11]. For the current analysis, we first analyzed these properties using a linear regression model that fit each soil parameter as a function of the cover-crop system, sampling time, and their interaction, which allows for the analysis of any date-specific effects. Soil nitrate and potassium concentration show significant differences between different sampling dates (P < 0.05). A significant interaction exists between the June sampling date and the legume cover crop system (CC and LM) on lime buffer capacity (LBC). No significant difference exists in soil phosphate concentration between either the sampling date or the cover crop system. The soil ammonium concentration result is excluded due to a strong violation of the linear regression assumption. An estimated marginal means test was then used to determine significant differences among the cover-crop systems (Fig 1). All cover crop systems had significantly lower LBC and higher soil zinc and nitrogen concentration compared to the NC control. The LM system also had significantly higher total organic carbon (TOC), soil pH, soil magnesium (Mg), potassium (K), calcium concentration (Ca), and base saturation percentage (BS) compared to the NC control (Table 1).

**Table 1:** Significance of the estimated marginal means test of soil chemical parameter differences among system groups.

Group1	Group2	LBC	K	Mg	Zn	Ca	N	TOC	BS	pH
NC	LM	**0.0023**	**0.0014**	**0.0054**	**0.0003**	**0.0319**	**0.0001**	**0.0453**	**0.0318**	**0.0332**
	CR	**0.0001**	**0.0207**	0.6697	**0.0001**	0.7827	**0.0001**	0.3897	0.4709	0.5935
	CC	**0.0001**	0.4778	0.1383	**0.0001**	0.1536	**0.0001**	0.4838	0.0743	0.1468
LM	CR	0.0085	0.1911	**0.0083**	0.5673	**0.0319**	**0.0080**	**0.0073**	0.0743	0.0606
	CC	0.1949	**0.0071**	0.1383	0.5982	0.4153	0.1377	0.1557	0.4709	0.3659
CR	CC	0.6303	0.0942	0.1977	0.8575	0.1202	0.2064	0.1557	0.2257	0.2884

TOC = total organic carbon. LBC = lime buffer activity (pH buffer capacity) p-values were adjusted by False Discovery Rate for multiple comparisons between groups.

When looking just among the different cover crop systems, LM had significantly higher TOC, N, Ca, and Mg levels compared to CR (Table 1), and significantly higher K compared to CC (Table 1).

Previous work [11] showed that cover crop systems also changed the soil surface physical properties (Table 2). Soils from cover crop systems were more porous compared to soils in the NC system. Soils from the LM system had much higher labile carbon content and lower density compared to the other systems.

**Table 2:** The mean surface properties of soil in each system sampled after 2018 harvest (four years after the start of experiment).

Treatment	BD	Porosity	Ksat	Water hold	POX-C
	g cm^–3 ^	%	cm h^–1^	cm^3^ cm^–3^	mg kg^–1^
NC	1.41 a	46.8 a	2.37 a	0.459 a	495 a
LM	1.25 b	52.7 b	8.05 b	0.462 a	603 b
CR	1.4 a	47.2 a	3.20 a	0.466 a	487 a
CC	1.36 a	48.7 a	3.25 a	0.460 a	544 ab

The significant letters were determined by Tukey Test. BD = bulk density, Ksat = surface saturated water permeability. Water hold = water holding capacity, POX-C = permanganate oxidizable carbon (labile carbon). Previously reported in [11].

### 3.2. Living mulch and cereal rye systems result in higher bacterial diversity and evenness

Soil microbiome samples were collected at 600 and 1400 growing degree days and shortly (<24 hours) after harvest of the primary crop (maize). Total microbial DNA was extracted, and the v4 region of 16S rRNA region was amplified with the Earth Microbiome Project primers for Illumina sequencing. After filtering out low-quality and organelle sequences, the amplicon sequence variant (ASV) table contained 35 samples and 1,683,598 high-quality reads with a median sequence depth of 41,739 per sample (S5 Fig and S2 Table in S1 File). The rarefaction analysis confirmed that subsampling to 19,000 reads effectively captured the diversity and most ASVs in the samples (S2 and S3 Figs in S1 File). The taxonomic distribution of ASV at the phylum level of all samples can be viewed in S6 Fig in S1 File.

**Fig 2:**
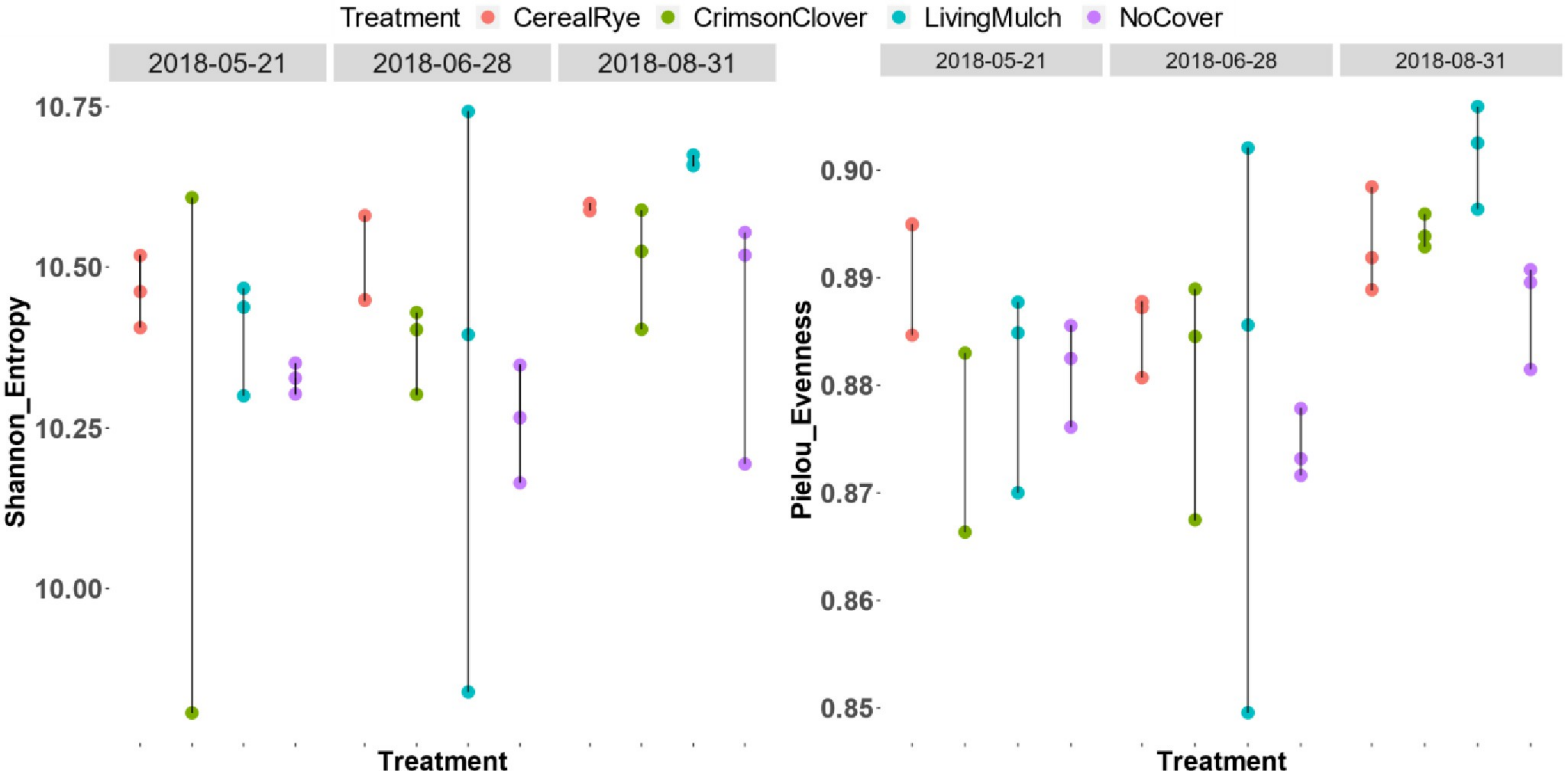
Pielou evenness and Shannon diversity of the microbiome community during the growing season in 2018. The Pielou evenness index ranges from 0 to 1, with more even (diverse) communities having higher values.

Alpha diversity (within-sample diversity) was calculated using the metrics of observed ASVs, Pielou evenness, and the Shannon diversity index. We then used linear regression to test the effect of cover crop and sampling time on these metrics. After excluding two outlier samples, both LM and CR show significantly higher diversity (Pielou evenness and Shannon index) compared to the NC control (Table 3), and the end-of-season (August) samples show higher diversity relative to the May samples (Fig 2). There were no significant interactions between systems and dates for the Shannon index, although there was a significant interaction between the LM system and the June sampling date for the Pielou evenness index (p-value = 0.020).

**Table 3:** Linear regression effect estimates and significance comparing Shannon index and Pielou index between system and sampling dates.

Sample Group	Pielou Estimate	p-value	Shannon Estimate	p-value
LivingMulch	0.0104	**0.00551**	0.200	**0.00721**
CerealRye	0.00898	**0.0121**	0.181	**0.00144**
CrimsonClover	0.00489	0.186	0.117	**0.0428**
2018-06-28	-0.00135	0.671	0.00212	0.965
2018-08-31	0.00983	**0.00347**	0.131	**0.00950**

Systems are relative to the NC control, and dates relative to the initial (May) sampling date.

We hypothesized that some of the differences in the bacterial community could be due to the different soil physical and/or chemical properties of the different systems. We tested this hypothesis using both linear regression and Spearman correlation to determine the relationship between soil chemical parameters and the alpha diversity (Pielou index) of the bacterial communities. The dataset was subset by sample date to avoid the dependence on time-series data. Since the soil was not sampled for chemical characteristics at harvest time, we used the previous sampling (July 18th) for the August microbiome samples. In the June sampling, only soil nitrogen, ammonia, and TOC were associated with the alpha diversity of the bacterial community, whereas by August, soil nitrogen, pH, BS, and magnesium showed an association with the community diversity (Table 4).

**Table 4:** The result of Spearman correlation and linear regression between soil chemical characteristics and Pielou evenness index.

Soil chemical variables	June: p-value linear regression	June: p-value Spearman correlation	August: p-value linear regression	August: p-value Spearman correlation
pH	0.308	0.172	**0. 0144**	**0.01**
BS	0.256	0.172	**0.0159**	**0.0145**
Ca	0.331	0.237	0.0559	**0.032**
Mg	0.129	0.087	**0.00866**	**0.013**
TOC	**0.0392**	**0.048**	0.153	0.059
N	**0.0191**	**0.007**	**7.96E-04**	**8.31E-04**
Amon	**0.0111**	**0.006**	0.0668	0.0627

BS = base saturation, Ca = soil calcium concentration, TOC = total organic matters, Amon = ammonium concentration, Mg = magnesium, N = total nitrogen concentration.

After correcting for multiple testing (n = 13), the association between soil nitrogen concentration and alpha diversity in August remains significant in both linear regression and Spearman correlation analysis (adjusted p-value = 0.01). The association of pH, Base saturation, and magnesium with alpha diversity in August remained significant in the Spearman correlation but became marginal in linear regression (0.05 < adjusted p-value <0.06).

### 3.3. Cover crops accommodate different bacterial communities compared to bare soils

Our main interest in this project was to compare each system to the no-cover control and, secondarily, to compare the LM system to other cover crop systems. As a first analysis, we calculated the beta diversity (between-sample diversity) among the samples using four different distance metrics: Bray-Curtis (a standard ecological metric) [40] weighted and unweighted UniFrac (which account for phylogenetic distances among the bacteria) [41], and Aitchison distance (a metric that accounts for the compositional nature of microbiome sequencing data) [42, 43]. With each of these metrics, the three cover crop systems show significant differences in the bacterial community compared to the NC control. (Table 5 and S7 Fig in S1 File). Differences among the cover crop systems depended on the metrics used. LM was significantly different from CR and CC based on the unweighted UniFrac distance (p = 0.00075 and 0.00372). It was not different based on the other three metrics (Bray-Curtis, Weighted UniFrac, and Aitchison; all adjusted P > 0.05). The high significance based on unweighted UniFrac implies that the differences between LM and other cover crops are likely due to rare taxa since unweighted UniFrac focuses on species presence/absence without regard to their abundance [41].

**Table 5:** Pairwise PERMANOVA results of beta diversity distances between systems and No cover control based on 1000 permutations.

Group 1	Group 2	Sample size	Adjusted p-value unweighted	Adjusted p-value weighted	Adjusted p-value Bray-Curtis	Adjusted p-value Aitchison
NoCover	LivingMulch	18	**0.002**	**0.004**	**0.002**	**0.004**
NoCover	CerealRye	18	**0.002**	**0.004**	**0.002**	**0.004**
NoCover	CrimsonClover	17	**0.002**	**0.004**	**0.002**	**0.004**
LivingMulch	CerealRye	18	**0.008**	0.089	0.063	0.449
LivingMulch	CrimsonClover	17	**0.037**	0.107	0.136	0.336
CerealRye	CrimsonClover	17	0.279	0.863	0.503	0.646

p-values were adjusted by False Discovery Rate for multiple comparisons between the groups.

Pairwise PERMANOVA was used to test samples at different time points within a system to confirm the observed differences do not result from different sampling dates. The May, June, and August samples are not significantly different from each other by weighted UniFrac, Bray Curtis, and Aitchison distance. (S3 Table in S1 File). Only one marginal significance was observed between May and August samples by unweighted UniFrac (adjusted p = 0.045), which can diminish if the ASV table is re-rarefied.

We next tested if the patterns seen in beta diversity were related to the soil’s physical and chemical parameters. Linear regression and Mantel tests based on the Spearman correlation were used to find possible associations between soil environmental factors and the soil microbiome community.

**Fig 3:**
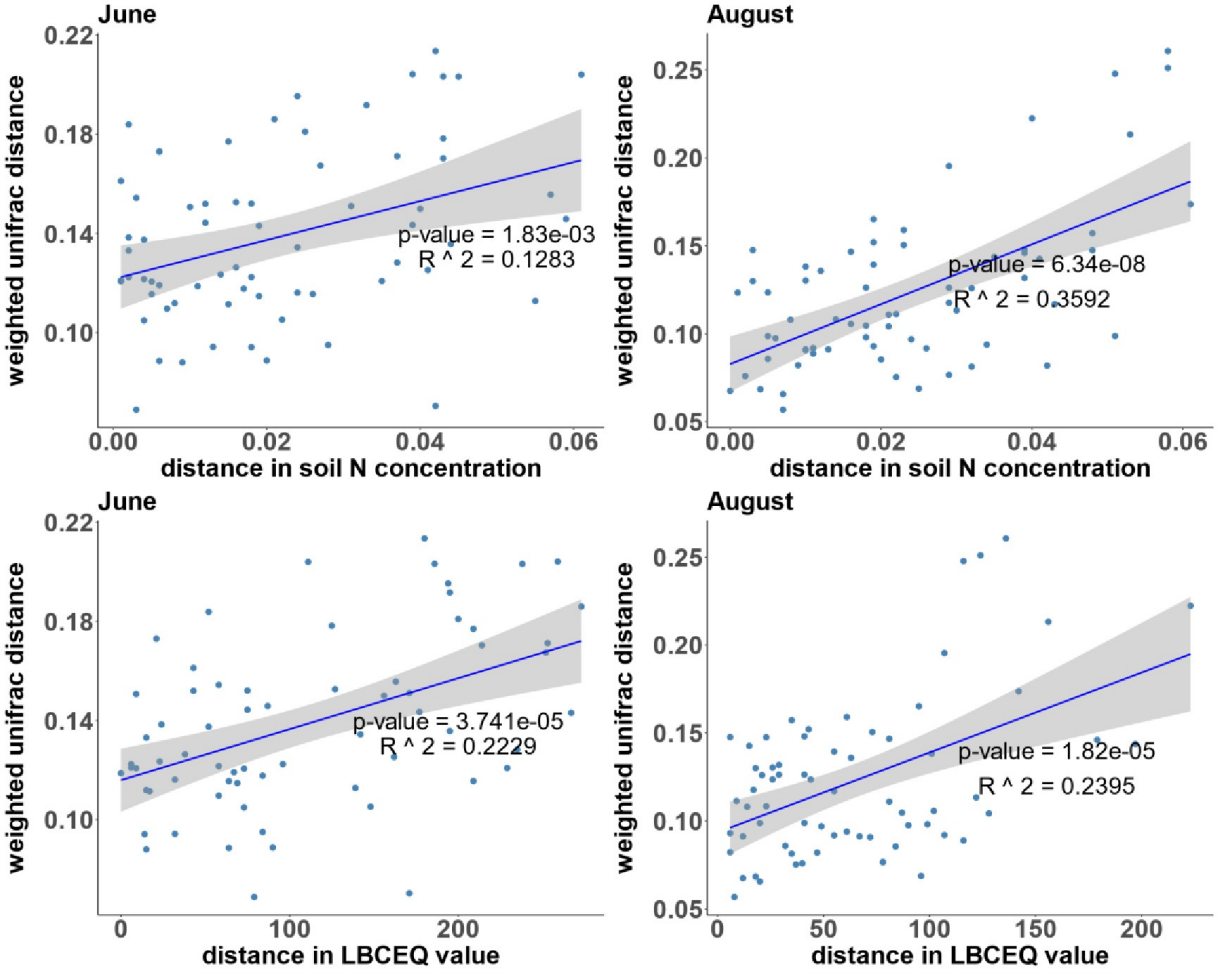
The linear regression between the distances in soil Nitrogen concentration (top) or Lime Buffer Capacity equilibrium value (bottom) against weighted UniFrac distance in June (left) and August (right) samples.

**Fig 4:**
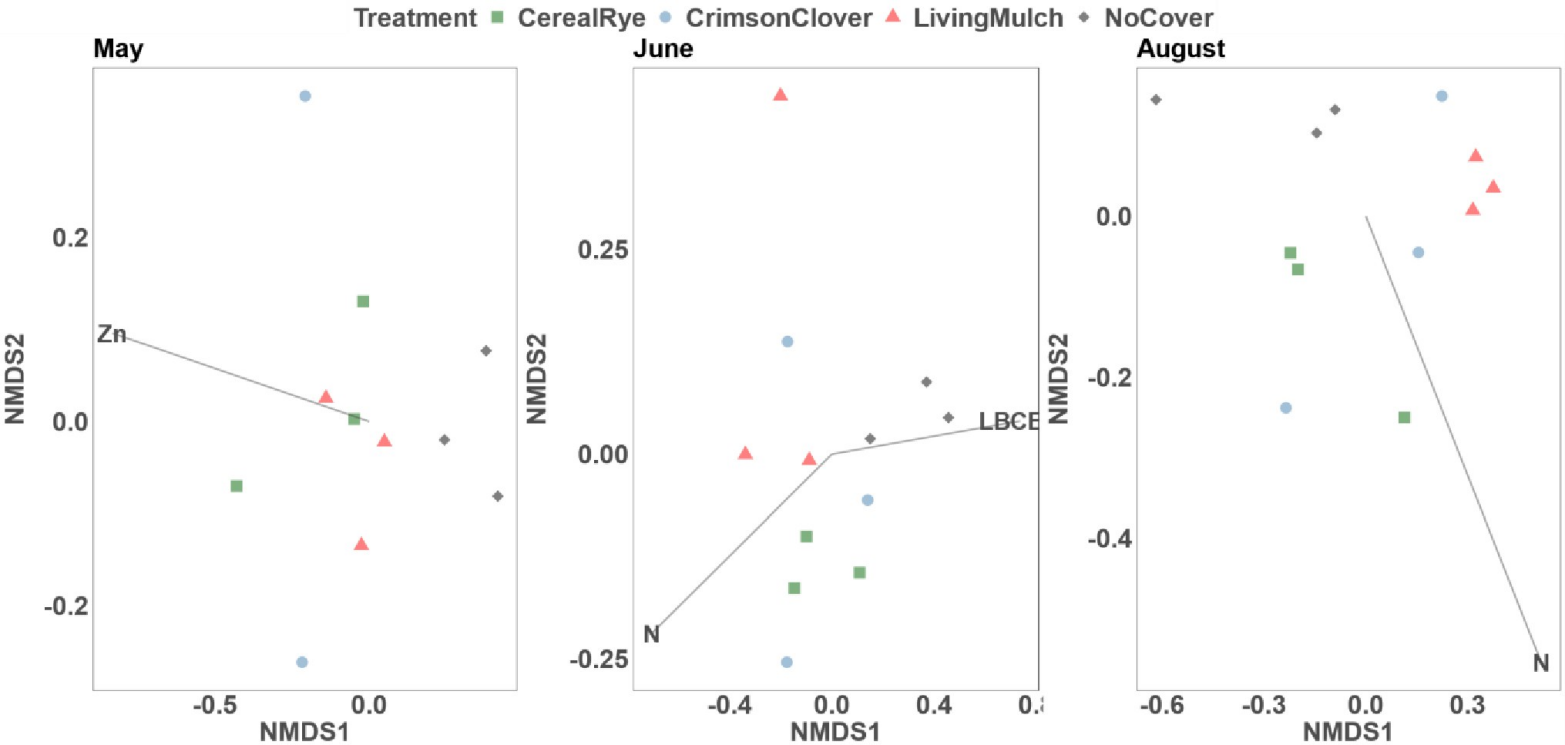
Non-metric multidimensional scaling of all samples based on Bray-Curtis distance. Soil environmental factors with a statistically significant association (p < 0.05) are shown. The lines with the soil parameter name show the direction of the (increasing) gradient, and the length of the arrow is proportional to the correlation between the variable and the ordination.

In the May samples, the difference in soil Zn concentration has a significant correlation with both UniFrac distances and the Bray-Curtis distances (Table 6). In June and August samples, the differences in soil nitrogen concentration and LBC values are associated with weighted and unweighted UniFrac distances (Fig 3 and S4 Table in S1 File). The association between soil nitrogen distances and weighted UniFrac distances became stronger in August, as evidenced by a stronger R^2^ value and smaller p-value. To visualize the correlation between soil chemical data and beta diversity, non-metric multidimensional scaling (NMDS) analysis based on Bray-Curtis distance was performed, shown in Fig 4.

**Table 6:** The raw p-values of a Mantel test (based on Spearman correlations) and linear regression comparing beta diversity distances to distances based on soil physical data.

	Weighted-Mantel:	Weighted lm:	Unweighted Mantel:	UnWeighted lm:	Bray-Curtis Mantel:	Bray-Curtis lm:	Aitchison Mantel	Aitchison lm
**Zn-May**	**0.004**	**5.29E-06**	**0.004**	**3.13E-04**	**0.005**	**6.05E-05**	**0.016**	**0.02**
**N-June**	**0.003**	**1.83E-03**	**0.034**	**1.93E-03**	0.177	0.0867	0.254	0.29
**LBC-June**	**0.008**	**3.74E-05**	**0.007**	**3.05E-06**	**0.023**	**3.57E-03**	**0.004**	**2.82E-03**
**N-August**	**0.003**	**6.34E-08**	**0.005**	**2.31E-07**	**0.011**	**4.61E-06**	**0.031**	**0.02**
**LBC-August**	**0.047**	**1.82E-05**	0.08	**4.35E-04**	0.11	**6.43E-04**	0.248	0.14

After correcting for multiple testing (n = 13), the associations between soil nitrogen, Lime buffer capacity, and two Unifrac distance were still significant by linear regression analysis (S4 Table in S1 File). However, only the association between soil nitrogen and Unifrac distance in August remained significant by Mantel test. The association between zinc concentration and beta diversity (weighted, unweighted Unifrac, and Bray-Curtis) was significant in linear regression analysis. The Mantel test only confirmed the significant association between Zinc concentration and Bray-Curtis distance and showed marginal significance with weighted and unweighted Unifrac.

### 3.4. Living mulch system causes the most differences in bacterial taxa

**Fig 5:**
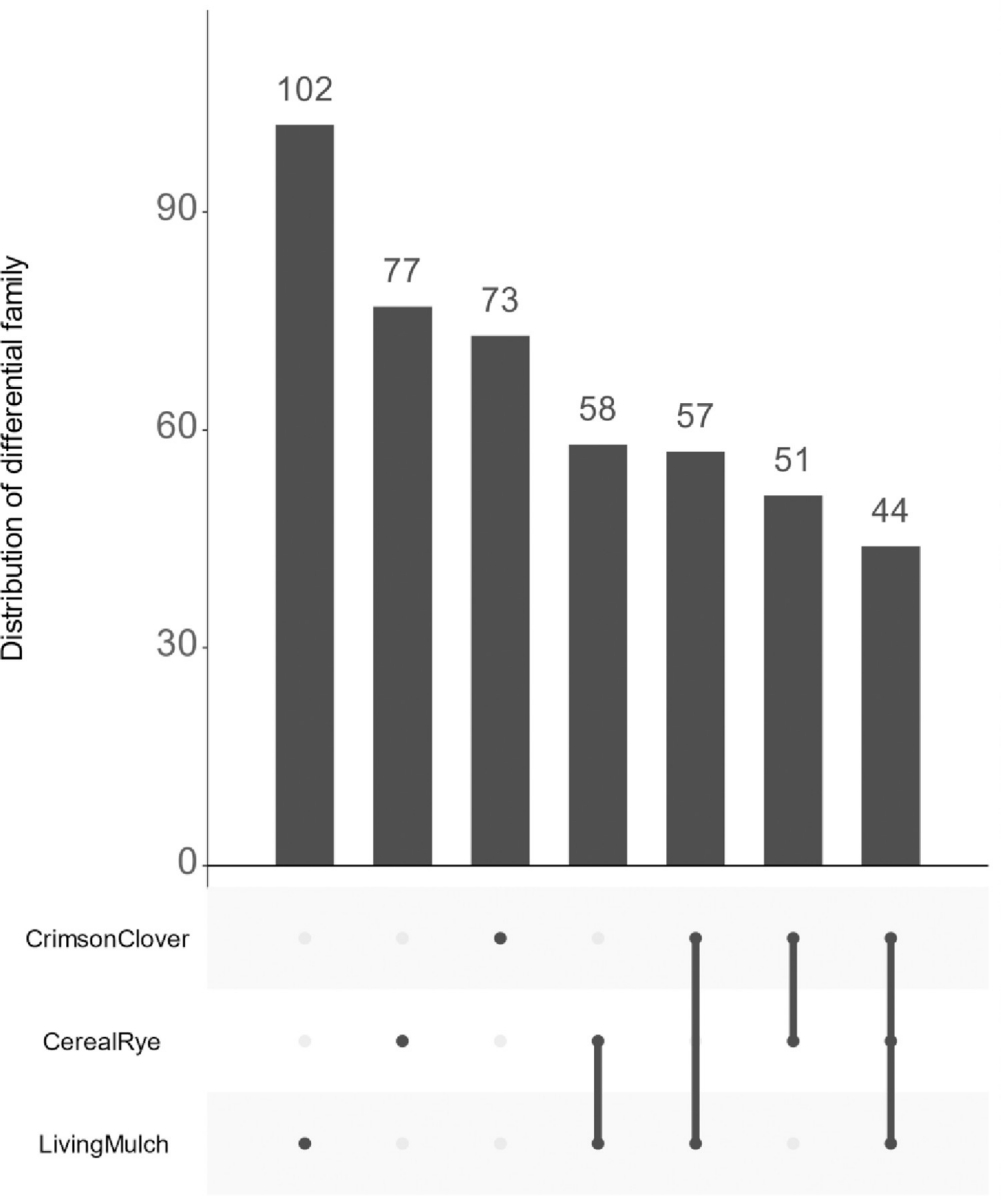
UpSet plot of the distribution of the differentially abundant bacterial taxa at the family taxonomic level of three cover crops system vs no-cover control. The bars at the lower left indicate the total number of differential taxa in each system, while the main panel shows the numbers shared across different combinations of systems.

We next determined which ASVs were significantly different among the cover-crop systems using DESeq2. Of the 12,142 taxa in the ASV Table, the LM system has 11045 differentially abundant ASVs (dfASV) compared to the NC control group at an adjusted p-value <0.05, the highest number among the three cover crop systems. CC has 761 dfASVs, and CR has 683 dfASVs compared to the NC control. To look at a broader taxonomic level, we also collapsed the ASV table at the family level (Fig 5). Among the 376 families, LM, CC, and CR have, respectively, 102, 73, and 77 differentially abundant families relative to the NC control.

Differences among the cover crops were much smaller. The LM system has only two differential families compared to CC and 12 differential families compared to CR (S5 Table in S1 File). No differential family was found between CR and CC systems.

Since the three cover crop systems were relatively similar, we identified the 44 differential families that were shared by all three of them relative to the NC control (S6 Table in S1 File). Of these 44, exactly half of them (22) show increasing abundances in cover crop systems, while the other half are less abundant than in the NC control. Vicinamibacteraceae, Pirellulaceae, and Gaiellales are the three most abundant families, respectively, with 2.97%, 2.51%, and 2.6% relative abundance of the community. The relative abundance of 18 moderately abundant differential bacterial family ranges from 0.1% ~ 2%. The rest of the differential family taxa have relative abundance less than 0.1%. Among the differential family, we found some characteristic bacteria for legume cover crops, such as *Rhizobiales*, *Frankiaceae*, and *Rhizobiaceae*.

We used linear mixed models to identify any associations between these families and soil chemical data. Among these 44 families, nine of them showed no association with soil chemical properties, 12 showed an association with LBC, and 32 showed an association with soil nitrogen concentration (S7 Table in S1 File). 10 families showed an association with both LBC and soil nitrogen concentration, but in opposite directions, indicating an antagonist effect between LBC and soil nitrogen on microbiome activity. When comparing differential families between CR, CC, and LM, only soil nitrogen concentration partially explained the differential pattern of families, while no significant association between taxa and LBC was found (S8 Table in S1 File).

## 4. Discussion

### 4.1. The living mulch system appears to benefit soil health

Relative to the no-cover control, the LM system increased not only total nitrogen concentration but also total organic carbon, especially labile carbon, potassium, and magnesium (Fig 1). Even though LM received much lower N fertilizer input (45 kg), no decrease in soil nitrate during the growing season was observed compared to other cover crop systems and NC control, and soil nitrogen concentration in the LM system was even higher than that of the CR system. This indicates there was vigorous nitrogen fixation by nitrogen-fixing bacteria, which is supported by a higher differential abundance of *Rhizobiaceae* in the LM system. Compared with CC (an annual legume cover crop), the perennial LM system showed a significant increase in potassium concentration, which is an important nutrient for plant growth (Fig 1). Moreover, the LM system is the only cover crop system to show significantly higher total organic carbon compared to NC, especially labile carbon concentration (Fig 1). LM also showed the most differentially abundant taxa with respect to NC, indicating that LM has a stronger impact on the soil microbiome community composition than CC and CR (Fig 5). However, this difference was small, given that the three cover crop systems were still relatively similar to each other.

The soil bacterial community under LM and CR systems has a higher Pielou evenness index, indicating a more heterogeneous distribution of microbes (Fig 2). A trend towards greater evenness was also seen in the microbiome community under CC, though it did not reach statistical significance (p = 0.186). Bacterial evenness has been identified as an important indicator of soil health, meaning the "capacity of a soil to function within ecosystem boundaries to sustain plant-animal productivity, maintain or enhance water and air quality, and support human health habitation” [44]. Pathogenic microbes face more intense competition in a balanced microbiome community and, thus are less likely to flourish [45, 46]. Moreover, a recent study found that a more diverse soil microbe community positively contributes to stronger plant-to-plant interactions and overall productivity in late-successional grassland species [47]. Although higher alpha diversity does not always mean a healthier condition [48–50], it does tend to be correlated with improved health. For example, a meta-analysis found that for 80% of the studies analyzed, higher diversity was positively associated with one or more microbially derived ecosystem functions [51].

### 4.2. Bacterial community changes likely due to soil physical and chemical properties

Plants can alter the soil physical and chemical environment, especially through root exudates, and these changes can directly or indirectly cause changes in the microbial community [52, 53]. Most of the differentially abundant bacteria in this study are associated with soil nitrogen concentration and lime buffer capacity (S8 and S9 Tables in S1 File), which also appeared to drive differences between the overall bacterial communities (Figs 3 and 4). The three most abundant differential taxa are *Gaiellales*, *Pirellulaceae*, and *Vicinamibacteraceae*, all of which correlated with total nitrogen concentration (S7 Table in S1 File). *Pirellulaceae* are anaerobic ammonia-oxidizing bacteria found in soils, wetlands, and marine sponges [54–56], and in our study, they were positively associated with total nitrogen concentration. The *Gaiellales* (negatively associated with total nitrogen) are strictly aerobic Actinobacteria that are catalase- and oxidase-positive, and some are predicted to reduce nitrate and perform CO_2_ fixation [57, 58]. The differential abundance of these bacteria might affect the nitrate turnover step in the soil nitrogen cycle. Moreover, *Nitrosotaleaceae*, an ammonia-oxidizing archaeon family that plays a major role in the soil nitrogen cycle, is more abundant in NC control compared to the three cover crop systems. Previous research suggests that ammonia-oxidizing archaea (AOA) actually prefer a low-ammonia environment [59, 60], which matches the lower ammonia concentration in the NC control relative to the cover crop systems. A meta-analysis of global field surveys also revealed that vegetated land also has fewer AOAs than bare soils [61], which is echoed in our experiment where the three cover crop treatments have less *Nitrososphaeria* than the control. This may be because of increased soil organic carbon, which was reported to inhibit the growth of some AOA species [62].

Moreover, the cover crop systems might change bacterial community composition by changing the soil oxygen environment. *Pirellulaceae* are anaerobic ammonia-oxidizing bacteria, and most of the *Pirellulaceae* here consist of the Pir4 lineage, which was found in various low-oxygen habitats [63]. Meanwhile, members of *Gaiellales* are strictly aerobic [57]. The lower abundance of *Gaiellales* and higher abundance of *Pirellulaceae* might indicate that cover crops created biological niches that favor anaerobic bacteria. Similarly, *Anaerolieae*, a class of fermentative bacteria that grow under strictly anaerobic conditions [64], are also preferentially abundant in three cover crop systems, while *Acetobacteraceae*, a family of strictly aerobic bacteria [65], decreased in abundance in all cover crop systems. No significant association between these two taxa and soil chemical properties was found. Our previous work showed that cover crop systems have higher CO_2_ emissions than NC control [66]. This higher respiration might deplete soil oxygen, which in turn promotes denitrification [67]. This hypothesis is supported by the higher N_2_O and NH_3_ emissions previously reported for the living mulch system [66].

Compared with NC control, cover crop systems show a much lower lime buffer capacity. Lime buffer activity measures the amount of acid-forming cations (such as H+, Al3+, Fe3+, and Mn2+) absorbed by the negatively charged soil particles [68]. Under wetting events such as rain and irrigation, these cations form acidic hydrated ions with water molecules and change the acidity of the soil microenvironment, which may explain the higher abundances of acidophilic bacteria (such as the *Acidobacteriota* phylum) in NC control. Moreover, members of *Acidimicrobiaceae* can perform ion-oxidizing or reduction metabolism under acidophilic conditions [65].

We also found that soil chemical characteristics were related to alpha diversity but that these relationships changed over time. In June samples, weak associations were found between the microbiome community’s evenness and soil nitrogen, ammonia, and total organic carbon (Table 4). In the August samples, however, we observed that the microbiome community’s evenness has a positive relationship with the soil nitrogen level, total organic carbon, magnesium concentration, calcium concentration, and base saturation value. No correlation was found in the May samples. One possible explanation is that it takes time for the soil microbiome community to respond to the addition of fertilizers on May 15th, so the sample on May 21st failed to capture such relationships. The reason for the association between soil chemical characteristics and alpha diversity is not known.

### 4.3. Potential role of soil aggregates in shaping bacterial communities of the different systems

One possible explanation for the different bacterial communities in these systems is the presence of more soil macroaggregates under cover cropping. Soil aggregates host the majority of the microbiome and are periodically connected through wetting events, which allow for the transfer of microbes, genetic material, and metabolites [69]. Higher organic matter and cation concentration also stabilize soil macroaggregates, which are known to favor specific groups of bacteria [70]. In previous work [11], the soils from living mulch on August 31^st^ had lower bulk density, but higher porosity, labile carbon content, and surface saturated permeability; these metrics indicate more loose, porous soils compared to other cover crop systems. Given that the water holding capacity is the same across the system, the increased porosity and permeability are likely to associate with macropores, which are not good at storing water [71–73]. Moreover, living mulch had much higher labile carbon, which is known to be significantly positively correlated with soil aggregate formation and stabilization [74].

Previous work demonstrates that bacterial groups such as *Acidobacteria*, *Actinobacteria*, *Bacteroidetes*, and *Cyanobacteria* are differentially more abundant in mega-aggregates under full residue retention systems compared to low residue retention systems [71]. Therefore, high organic residue from the living mulch system and better stability of soil aggregates might create habitats with nutrients that favor some bacterial groups and thus shift the bacterial community balance. This hypothesis still needs further confirmation, and it will be investigated in future work.

## 5. Conclusion

Meeting future food security needs will require sustainable and innovative agriculture solutions to increase food production while maintaining environmental health. In this study, we showed that cover crops—and especially living mulch—significantly increased the diversity and evenness of the soil bacterial community, traits that have often been shown to be positively related to the health of the soil community. Although this living mulch system has lower maize yield than conventional production, the much lower cost of inputs can actually make it more profitable to growers than conventional production. Moreover, further optimization of fertilizer input and field management could avoid losses in yield. (For example, one study reported that a reduction in cornrow width led to more vigorous corn growth and higher yield than conventional corn farming [75]). Therefore, living mulch appears to represent a promising perennial cover crop option for the agriculture industry in areas with mild winters by lowering nitrogen fertilizer input, increasing economic returns, providing perennial soil erosion protection, and enhancing soil health and the soil bacterial community.

## Supporting information

S1 Appendix Living_mulch_primer_sequences. (XLSX)

S1 File Additional files for living mulch paper. (DOCX)
