# Supplementary material for: A perennial living mulch system fosters a more diverse and balanced soil bacterial community
Source: PLoS One. 2023 Aug 29;18(8):e0290608. doi: 10.1371/journal.pone.0290608 (PMC10464973; doi:10.1371/journal.pone.0290608)
Supplement: S1 File — (DOCX) [file pone.0290608.s002.docx]

Supplementary Data


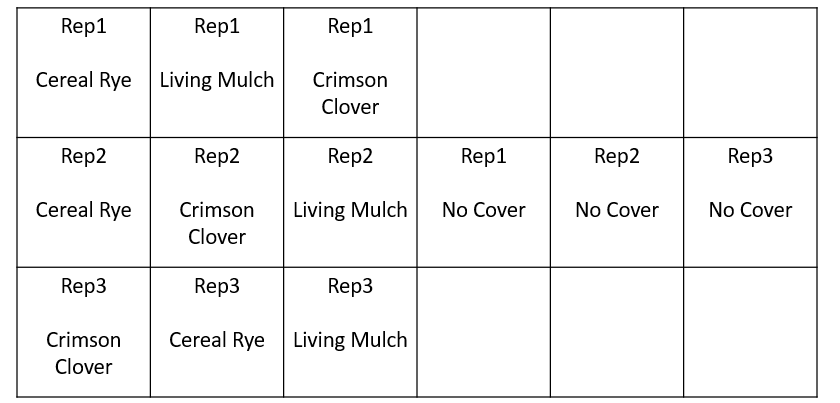


**Supplement Figure 1.** **Experiment field layout at the J. Phil Campbell Research and Education Center in Watkinsville, GA (33°52 09.5˝ N 83°26´59.8˝ W; 219 m elevation).** Each treatment has three biological replicates. No Cover control were separated from cover crop treatments in another block.


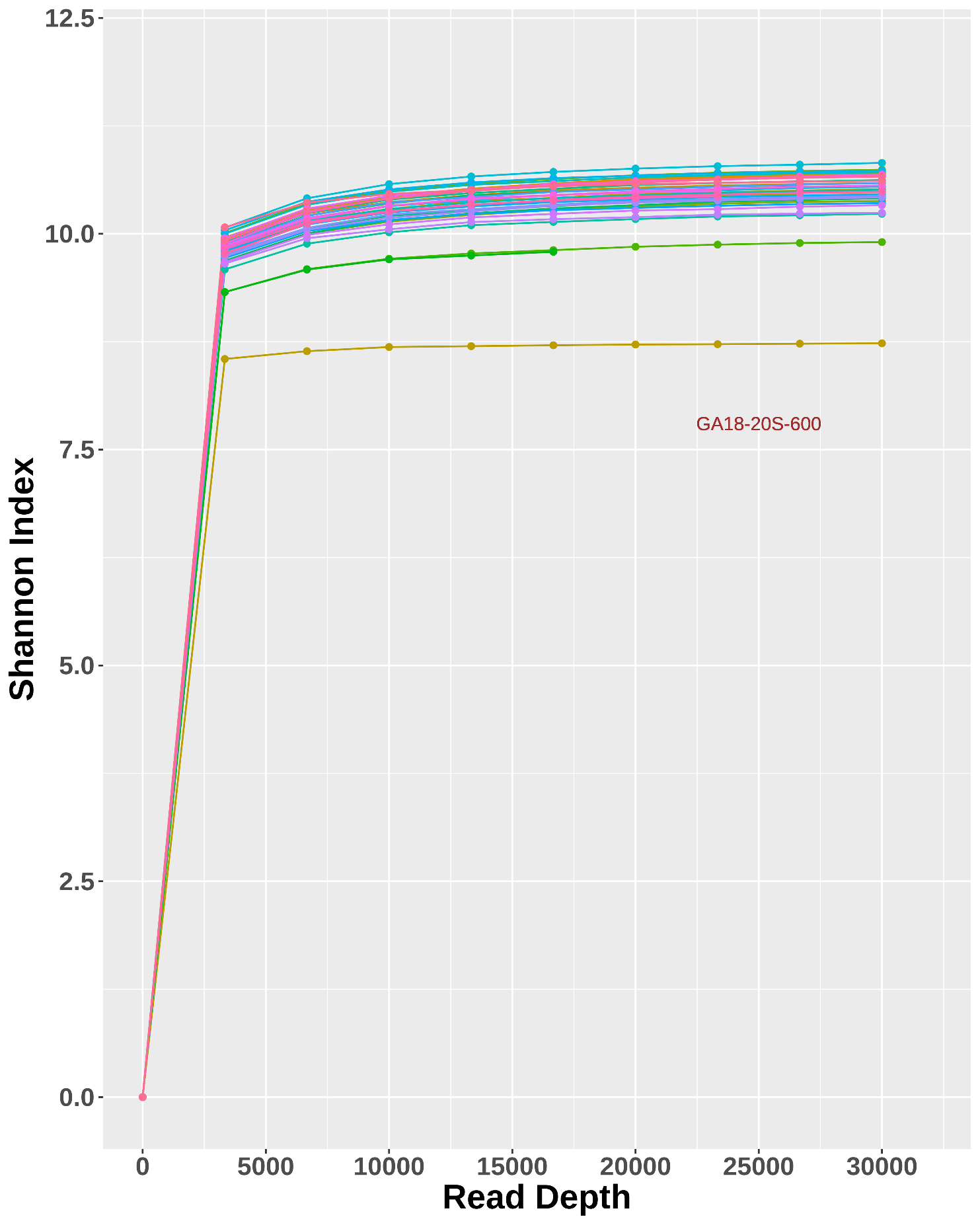


**Supplement Figure 2.** Rarefaction curve of samples, showing the Shannon index. Samples plateau around 5-10k reads, so the chosen sequence depth of 19k should capture the diversity of the communities


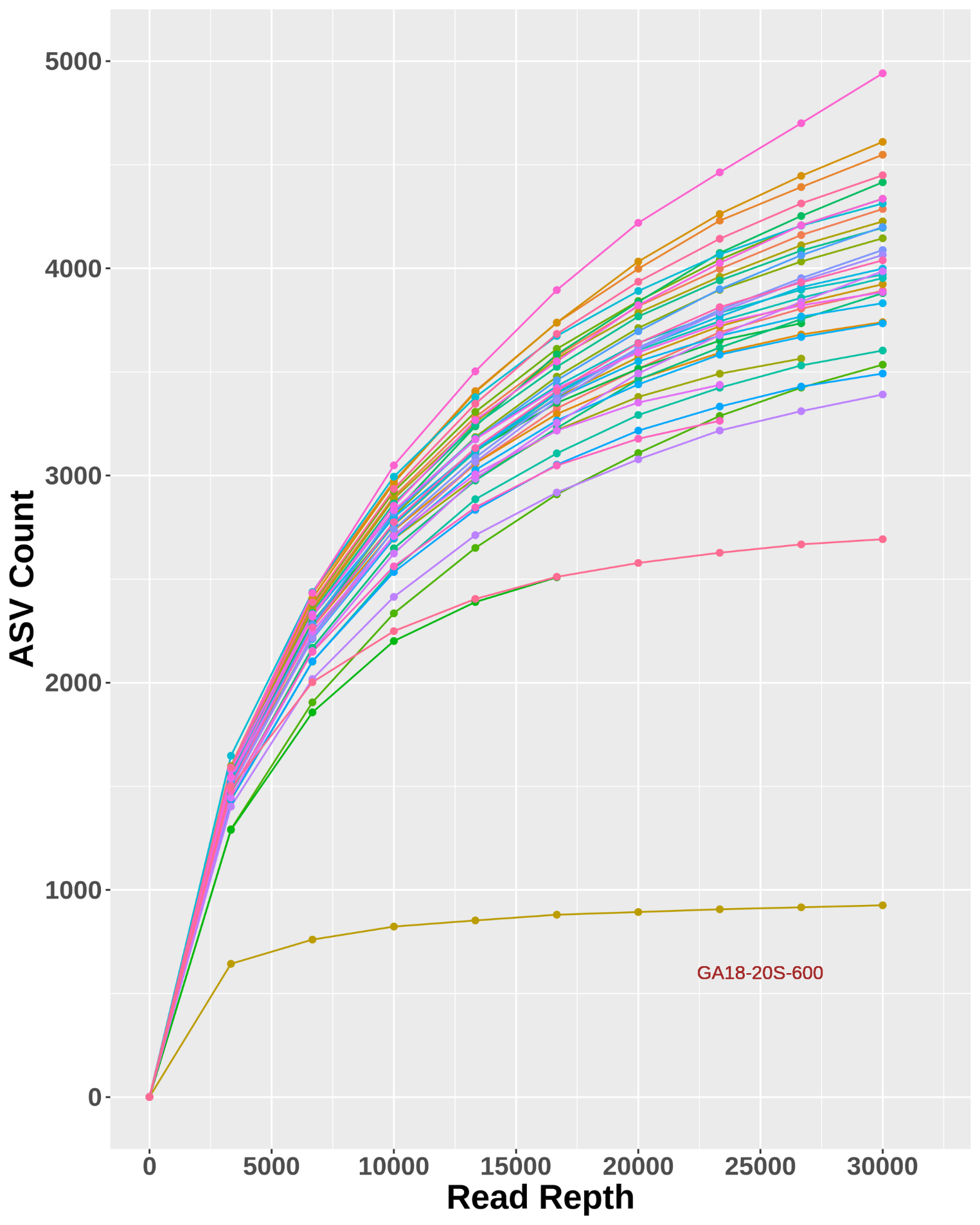


**Supplementary Figure 3** Rarefaction curve of the number of observed ASV of samples. GA18-20S-600 has a much lower diversity from all the other samples and therefore excluded from downstream analysis. The chosen sequence depth of 19000 should capture most ASVs in each sample

| ASSESSMENT OF THE LINEAR MODEL ASSUMPTIONS  USING THE GLOBAL TEST ON 4 DEGREES-OF-FREEDOM:  Level of Significance = 0.05 | | | |
| --- | --- | --- | --- |
| Call:  gvlma::gvlma (x = lm_treatment_date_relation_shannon) | | | |
|  | Value | p-value | Decision |
| Global Stat | 37.2313 | 1.614e-07 | Assumptions NOT satisfied! |
| Skewness | 14.0102 | 1.818e-04 | Assumptions NOT satisfied! |
| Kurtosis | 22.7089 | 1.885e-06 | Assumptions NOT satisfied! |
| Link Function | 0.0264 | 8.709e-01 | Assumptions acceptable. |
| Heteroscedasticity | 0.4858 | 4.858e-01 | Assumptions acceptable. |

**Supplementary Table 1** Assumption test of linear regression model using gvlma function in R.

Exclusion of several outliers (see Methods) results in all assumptions being satisfied


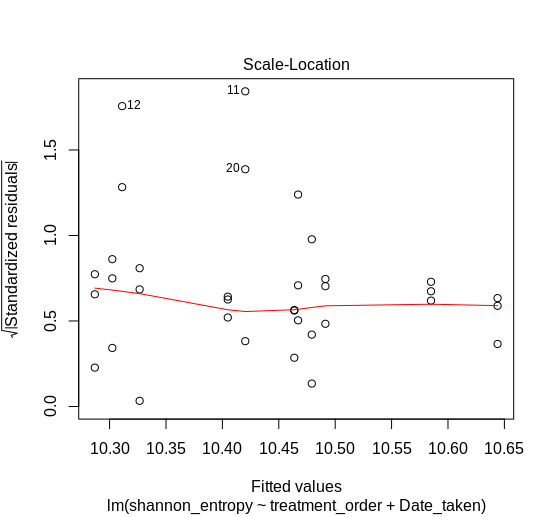

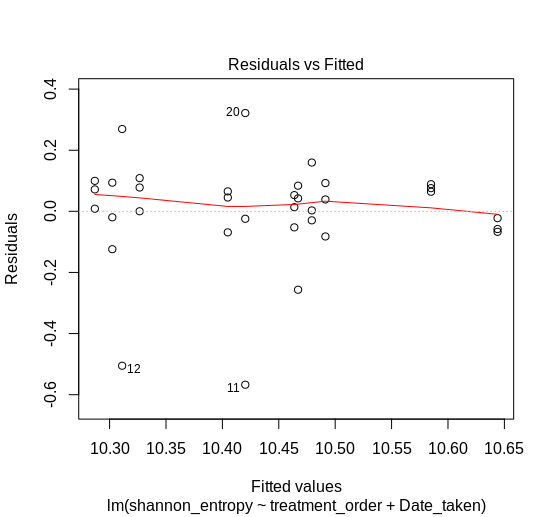


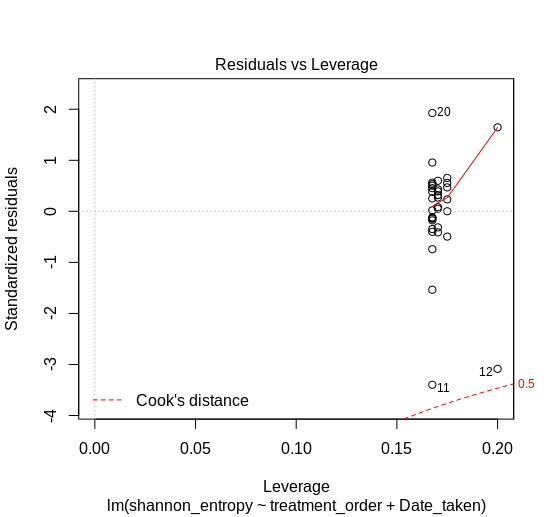

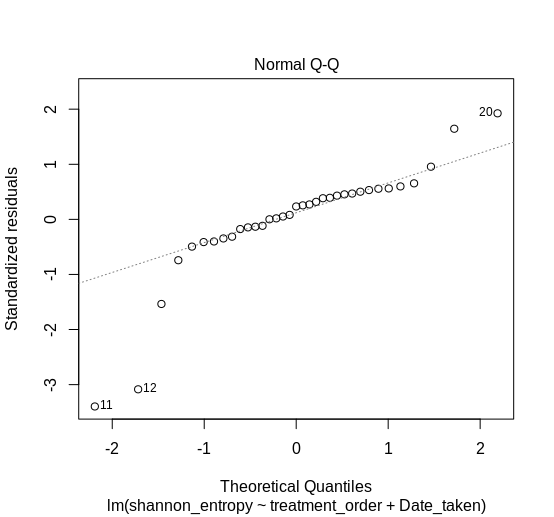


**Supplementary Figure 4** Graph of residue and linear relationship of original linear models. The 11th and 12th samples are GA18-21S-LM1400 and GA18-22S-600


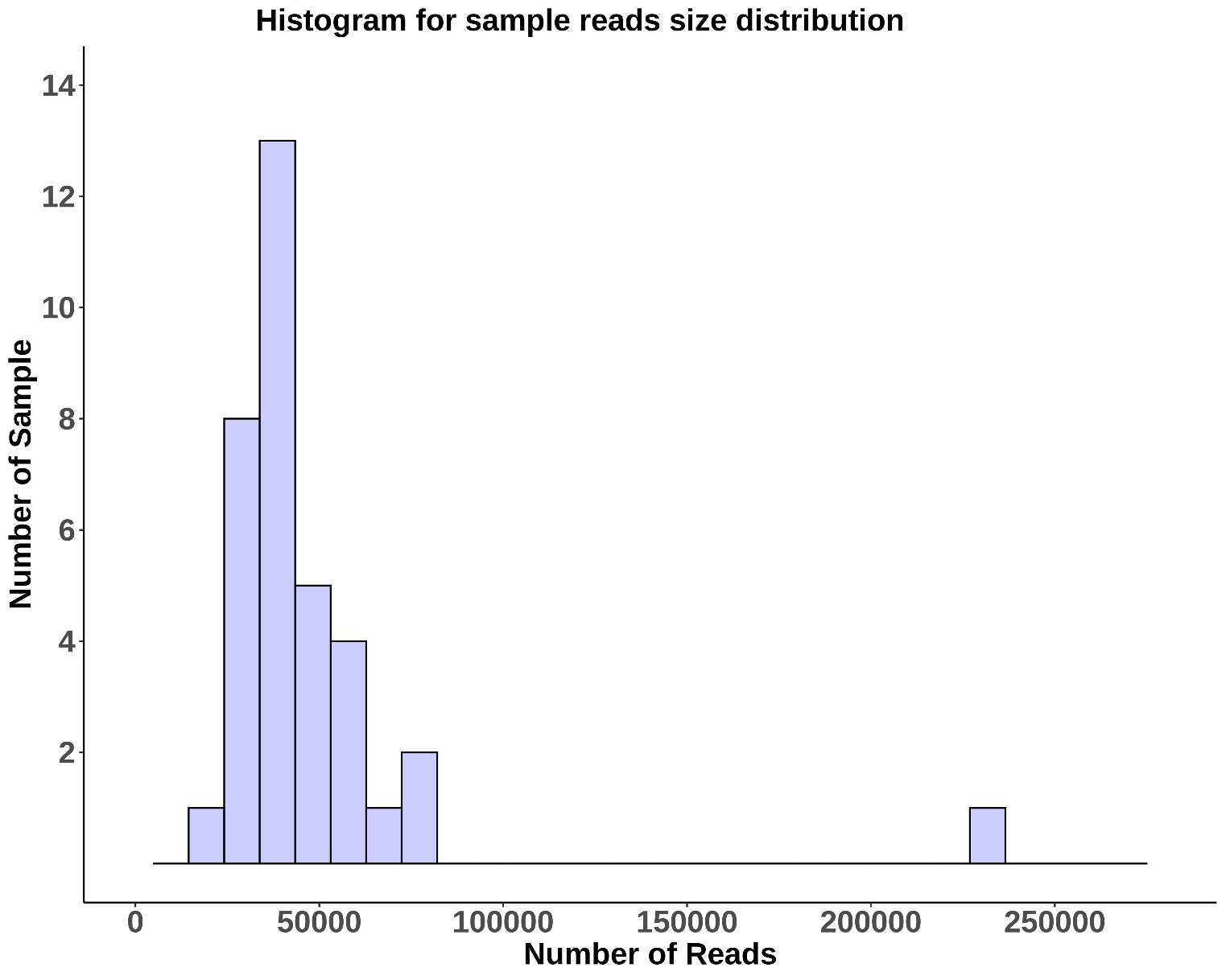


**Supplementary Figure 5** Read depth values for the 35 samples in the final dataset. Most samples cluster around 30k-40k reads, with one outlier at ~250k

| Metric | Value |
| --- | --- |
| Number of samples | 35 |
| Number of ASV | 12142 |
| Total frequency | 1683598 |
| Minimum frequency | 19321 |
| 1^st^ quartile | 33004 |
| Median frequency | 41739 |
| 3^rd^ quartile | 52584.5 |
| Maximum frequency | 233878 |
| Mean frequency | 48102.8 |

**Supplementary Table 2** Summary statistics of the ASV table after filtering.


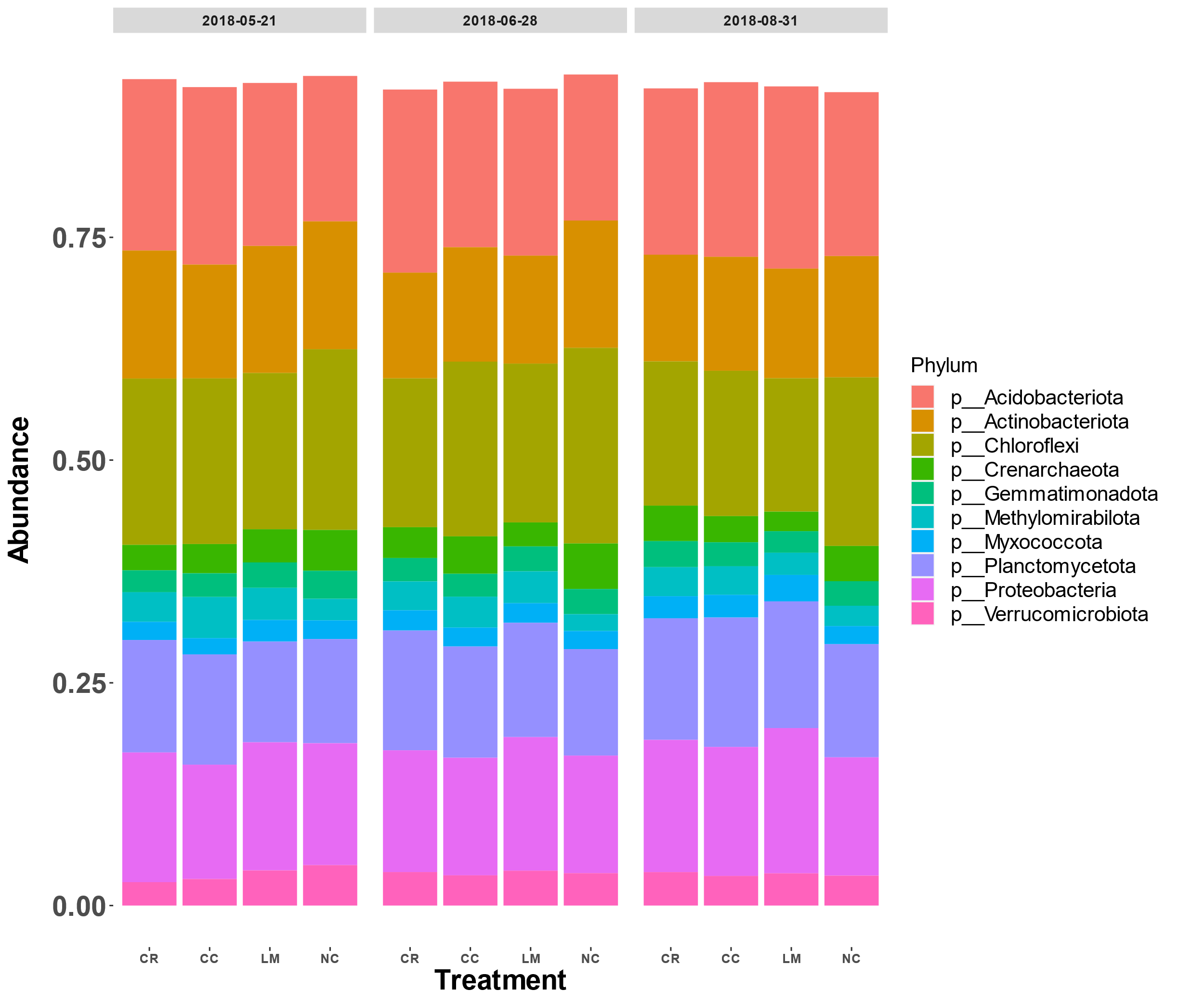


**Supplementary Figure 6** The taxonomic distribution of top 10 abundant taxa at phylum level of each system separated by sample date.


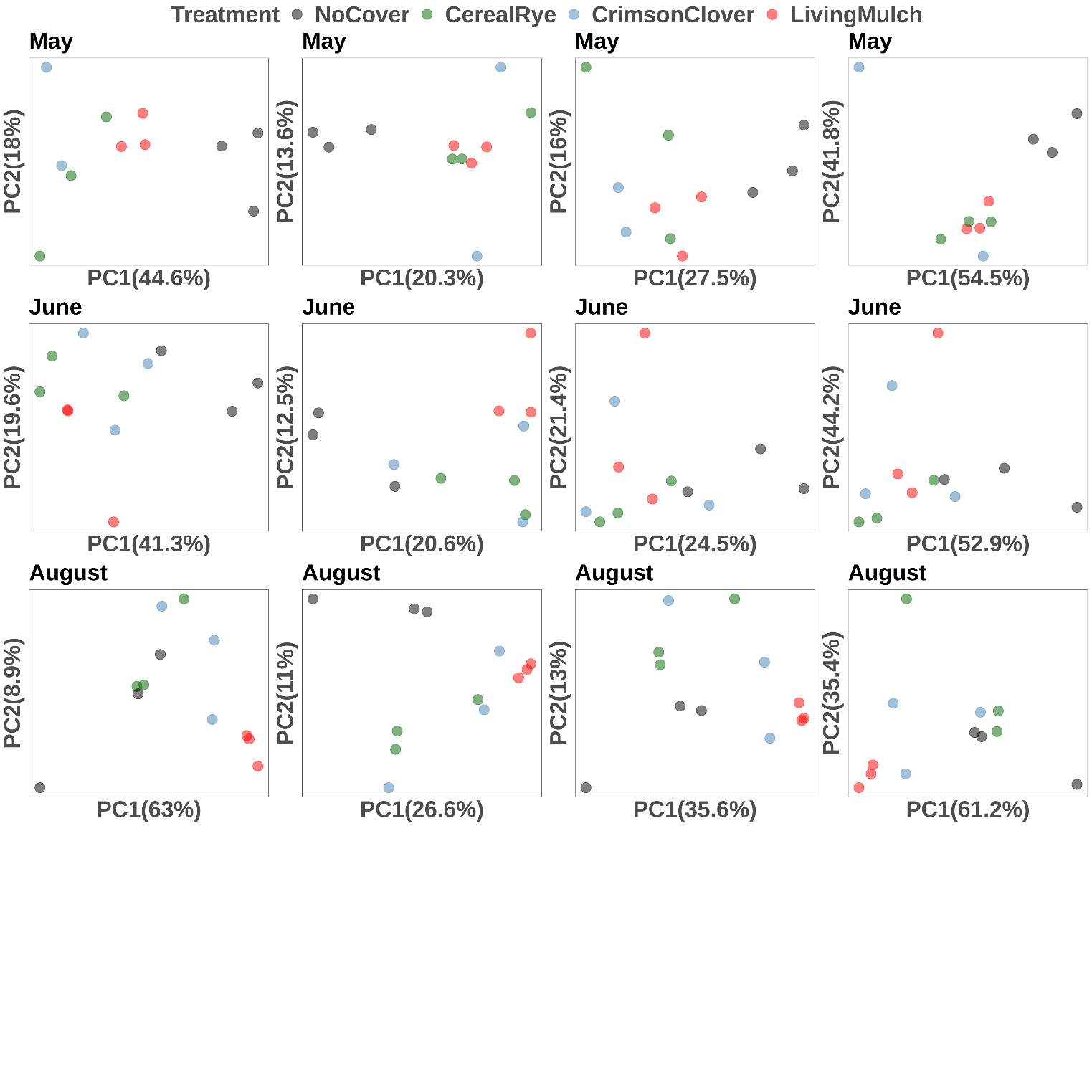


**Supplementary Figure 7** The principal coordinates graph of the bacterial communities based on three beta diversity: weighted unifrac(left), unweighted unifrac(middle left), Bray Curtis(middle right),Aitchison distance (right).

| Group 1 | Group 2 | Sample size | Weighted:  q-value | Unweighted:  q-value | Bray-Curtis:  q-value | Aitchison  q-value |
| --- | --- | --- | --- | --- | --- | --- |
| 2018/5/21 | 2018/6/28 | 23 | 0.693 | 0.382 | 0.742 | 0.590 |
| 2018/5/21 | 2018/8/31 | 23 | 0.186 | 0.045 | 0.201 | 0.204 |
| 2018/6/28 | 2018/8/31 | 24 | 0.336 | 0.382 | 0.621 | 0.590 |

**Supplementary Table 3** Pairwise PERMANOVA results of comparing sampling times based on weighted Unifrac, unweighted Unifrac, and Bray-Curtis distance matrices

|  | Weighted-Mantel: | Weighted lm: | Unweighted  Mantel: | UnWeighted  lm: | Bray-Curtis Mantel: | Bray-Curtis  lm: | Aitchison  Mantel | Aitchison  lm |
| --- | --- | --- | --- | --- | --- | --- | --- | --- |
| Zn-May | 0.065 | **6.88E-05** | 0.065 | **4.06E-03** | **0.013** | **7.87E-04** | 0.182 | 0.11 |
| N-June | 0.195 | **1.19E-02** | 0.149 | **1.25E-02** | 0.611 | 0.564 | 0.69 | 0.746 |
| LBC-June | 0.078 | **4.86E-04** | 0.065 | **3.95E-05** | 0.26 | **0.0464** | 0.36 | **0.036** |
| N-August | **0.039** | **8.24E-07** | **0.039** | **3.05E-06** | 0.117 | **5.98E-05** | 0.265 | 0.149 |
| LBC-August | 0.225 | **1.18E-04** | 0.65 | **2.82E-03** | 0.507 | **4.17E-03** | 0.37 | 0.33 |
|  |  |  |  |  |  |  |  |  |

**Supplementary Table 4** The adjusted p-values by false discovery rate of a Mantel test (based on Spearman correlations) and linear regression comparing beta diversity distances to distances based on soil physical data.

| Taxa | Rank | log2FoldChange | Abundance |
| --- | --- | --- | --- |
|  |  | Living Mulch vs Cereal Rye |  |
| Xanthomonadaceae | Family | 0.71 | 0.0023 |
| Rhizobiaceae | Family | 0.87 | 0.0022 |
| Polyangiales | Order | 0.83 | 0.0017 |
| Verrucomicrobiaceae | Family | 1.49 | 0.0015 |
| Nocardiaceae | Family | 1.28 | 0.0013 |
| Sandaracinaceae | Family | 1.51 | 0.0011 |
| Chloroflexaceae | Family | -1.99 | 0.0007 |
| Kallotenuales | Order | -1.32 | 0.0005 |
| Flavobacteriaceae | Family | 1.62 | 0.0004 |
| Methylophilaceae | Family | 3.69 | 0.0002 |
| Nostocaceae | Family | -4.93 | 0.0001 |
| Polyangia | Class | 3.66 | 0.0001 |
|  |  | Living Mulch vs Crimson Clover | |
| Verrucomicrobiaceae | Family | 1.21 | 0.0015 |
| Cryptosporangiaceae | Family | -4.55 | 0.00005 |

**Supplementary table 5** Differential abundances of taxa between Living Mulch, Cereal Rye, and Crimson Clover system. The abundance is the relative abundance of the taxa in total community abundance. The significance of result was evaluated by adjusted p-value (<0.05) using False Discovery Rate (Benjamini & Hochberg, 1995) correcting for multiple testing.

| Taxa | Rank | log2FoldChange | log2FoldChange | log2FoldChange | Relative Abundance | |  |
| --- | --- | --- | --- | --- | --- | --- | --- |
|  |  | Living Mulch | Crimson Clover | Cereal Rye |  | |  |
|  |  |  |  |  |  | |  |
|  |  | High abundance group | | |  | |  |
| Gaiellales | Order | -0.6 | -0.58 | -0.59 | 0.0260 | | |
| Pirellulaceae | Family | 0.41 | 0.41 | 0.38 | 0.0251 | | |
| Vicinamibacteraceae | Family | 0.71 | 0.57 | 0.52 | 0.0297 | | |
|  |  | Middle abundance group | | |  | |  |
| Chloroflexi | Phylum | -0.73 | -0.66 | -0.7 | 0.0193 |  |  |
| Anaerolineae | Class | 0.83 | 1.34 | 1.11 | 0.0026 |  |  |
| Ktedonobacteria | Class | -1.1 | -0.64 | -0.76 | 0.0114 |  |  |
| Vicinamibacteria | Class | 0.51 | 0.47 | 0.47 | 0.0099 |  |  |
| Acidobacteriales | Order | -0.95 | -1.26 | -1.03 | 0.0108 |  |  |
| Elsterales | Order | -0.73 | -0.98 | -0.81 | 0.0094 |  |  |
| Ktedonobacterales | Order | -1.87 | -1.75 | -1.69 | 0.0051 |  |  |
| Rokubacteriales | Order | 1.26 | 1.58 | 1.33 | 0.0030 |  |  |
| Tepidisphaerales | Order | -0.6 | -0.43 | -0.45 | 0.0137 |  |  |
| Acetobacteraceae | Family | -0.63 | -0.49 | -0.52 | 0.0037 |  |  |
| Acidothermaceae | Family | -1.2 | -1.18 | -1.19 | 0.0049 |  |  |
| Bryobacteraceae | Family | -0.96 | -0.99 | -0.8 | 0.0043 |  |  |
| Comamonadaceae | Family | 0.96 | 0.83 | 0.54 | 0.0036 |  |  |
| Geodermatophilaceae | Family | -1.03 | -0.65 | -0.75 | 0.0028 |  |  |
| Isosphaeraceae | Family | -0.78 | -0.45 | -0.59 | 0.0122 |  |  |
| Ktedonobacteraceae | Family | -1.48 | -1.63 | -1.47 | 0.0134 |  |  |
| Rhizobiales_Incertae_Sedis | Family | 0.45 | 0.47 | 0.44 | 0.0035 |  |  |
| Solibacteraceae | Family | -0.74 | -0.81 | -0.61 | 0.0032 |  |  |
|  |  | Low abundance class | | |  | |  |
| Acidobacteriota | Phylum | 1.04 | 1.38 | 1.26 | 0.0015 |  |  |
| Acidobacteriota | Phylum | 1.14 | 1.05 | 1.2 | 0.0004 |  |  |
| WPS-2 | Phylum | -1.72 | -2.85 | -1.76 | 0.0008 |  |  |
| Gammaproteobacteria | Class | 1.38 | 1.04 | 1.47 | 0.0010 |  |  |
| Ktedonobacteria | Class | -2.19 | -3.32 | -1.72 | 0.0009 |  |  |
| Ktedonobacteria | Class | -4.58 | -5.11 | -3.45 | 0.0001 |  |  |
| Phycisphaerae | Class | 1.96 | 2.14 | 1.83 | 0.0002 |  |  |
| Planctomycetes | Class | 1.18 | 0.96 | 1.08 | 0.0006 |  |  |
| Acidimicrobiaceae | Family | -0.85 | -1.26 | -0.9 | 0.0004 |  |  |
| Acidobacteriaceae | Family | -1.11 | -1.46 | -1.18 | 0.0023 |  |  |
| Acidobacteriae | Family | 1.79 | 1.29 | 1.25 | 0.0003 |  |  |
| Chthonomonadaceae | Family | -1.45 | -1.08 | -0.82 | 0.0011 |  |  |
| Devosiaceae | Family | 1.57 | 1.21 | 1.04 | 0.0009 |  |  |
| Entotheonellaceae | Family | 1.24 | 1.46 | 1.5 | 0.0004 |  |  |
| Fimbriimonadaceae | Family | 1.43 | 1.84 | 2 | 0.0003 |  |  |
| Frankiaceae | Family | -1.24 | -0.84 | -1.08 | 0.0022 |  |  |
| Microbacteriaceae | Family | 1.68 | 1.5 | 1.06 | 0.0010 |  |  |
| Nitrosotaleaceae | Family | -2.6 | -1.88 | -1.82 | 0.0019 |  |  |
| Phycisphaeraceae | Family | 1.12 | 0.91 | 0.92 | 0.0009 |  |  |
| Pseudomonadaceae | Family | 2.2 | 1.91 | 1.58 | 0.0009 |  |  |
| Rhizobiaceae | Family | 1.65 | 1.33 | 0.78 | 0.0022 |  |  |
| Rubinisphaeraceae | Family | 1.12 | 0.79 | 0.98 | 0.0022 |  |  |
| Verrucomicrobiaceae | Family | 2.24 | 1.03 | 0.75 | 0.0015 |  |  |

**Supplementary table 6** Differential abundances of taxa between three cover crop treatment and No Cover crop (reference level) The abundance is the relative abundance of the taxa in total community abundance. The significance of result was evaluated by adjusted p-value (<0.05) using False Discovery Rate (Benjamini & Hochberg, 1995) correcting for multiple testing.

| Taxa | Rank | metadata | Correlation_LBC | Correlation_N | Abundance |
| --- | --- | --- | --- | --- | --- |
|  |  |  |  |  |  |
|  |  | High abundance group | |  |  |
|  |  |  | |  |  |
| Gaiellales | Order | N |  | - | 0.0261 |
| Pirellulaceae | Family | N |  | + | 0.0252 |
| Vicinamibacteraceae | Family | N |  | + | 0.0297 |
|  |  | Mid abundance group | |  |  |
|  |  |  | |  |  |
| Chloroflexi | Phylum | LBCEQ N | + | - | 0.0193 |
| Anaerolineae | Class |  |  |  | 0.0026 |
| Ktedonobacteria | Class | N |  | - | 0.0114 |
| Vicinamibacteria | Class | N |  | + | 0.0099 |
| Acidobacteriales | Order | N |  | - | 0.0108 |
| Elsterales | Order |  |  |  | 0.0094 |
| Ktedonobacterales | Order | LBCEQ N | + | - | 0.0051 |
| Rokubacteriales | Order | N |  | + | 0.0030 |
| Tepidisphaerales | Order |  |  |  | 0.0137 |
| Acetobacteraceae | Family |  |  |  | 0.0037 |
| Acidothermaceae | Family | LBCEQ N | + | - | 0.0049 |
| Bryobacteraceae | Family | LBCEQ N | + | - | 0.0043 |
| Comamonadaceae | Family | N |  | + | 0.0036 |
| Geodermatophilaceae | Family |  |  |  | 0.0028 |
| Isosphaeraceae | Family | N |  | - | 0.0122 |
| Ktedonobacteraceae | Family | LBCEQ N | + | - | 0.0134 |
| Rhizobiales | Family | N |  | + | 0.0035 |
| Solibacteraceae | Family | N |  | - | 0.0032 |
|  |  |  |  |  |  |
|  |  | Low abundance class | |  |  |
|  |  |  | |  |  |
| Acidobacteriota | Phylum | LBCEQ | - |  | 0.0015 |
| Acidobacteriota | Phylum | N |  | + | 0.0004 |
| WPS-2 | Phylum | N |  | - | 0.0008 |
| Gammaproteobacteria | Class |  |  |  | 0.0010 |
| Ktedonobacteria | Class | LBCEQ N | + | - | 0.0009 |
| Ktedonobacteria | Class |  |  |  | 0.0001 |
| Phycisphaerae | Class | LBCEQ N | - | + | 0.0002 |
| Planctomycetes | Class | N |  | + | 0.0006 |
| Acidimicrobiaceae | Family | LBCEQ N | + | - | 0.0004 |
| Acidobacteriaceae | Family | LBCEQ N | + | - | 0.0023 |
| Acidobacteriae | Family | N |  | + | 0.0003 |
| Chthonomonadaceae | Family | N |  | - | 0.0011 |
| Devosiaceae | Family | N |  | + | 0.0009 |
| Entotheonellaceae | Family | LBCEQ | - |  | 0.0004 |
| Fimbriimonadaceae | Family |  |  |  | 0.0003 |
| Frankiaceae | Family | N |  | - | 0.0022 |
| Microbacteriaceae | Family | N |  | + | 0.0010 |
| Nitrosotaleaceae | Family | LBCEQ N | + | - | 0.0019 |
| Phycisphaeraceae | Family |  |  |  | 0.0009 |
| Pseudomonadaceae | Family | N |  | + | 0.0009 |
| Rhizobiaceae | Family | N |  | + | 0.0022 |
| Rubinisphaeraceae | Family | N |  | + | 0.0022 |
| Verrucomicrobiaceae | Family | N |  | + | 0.0015 |
|  |  |  |  |  |  |

**Supplementary Table 7** The association between differential family taxa of three cover crop treatment compared No Cover crop (reference level) and soil chemical The abundance is the relative abundance of the taxa in total community abundance.

| Taxa | Rank | metadata | Correlation_LBCEQ | Correlation_N | Abundance |
| --- | --- | --- | --- | --- | --- |
|  |  | Living Mulch vs Cereal Rye | | |  |
| Xanthomonadaceae | Family | N |  | + | 0.0023 |
| Rhizobiaceae | Family | N |  | + | 0.0022 |
| Polyangiales | Order |  |  |  | 0.0017 |
| Verrucomicrobiaceae | Family | N |  | + | 0.0015 |
| Nocardiaceae | Family |  |  |  | 0.0013 |
| Sandaracinaceae | Family | N |  | + | 0.0011 |
| Chloroflexaceae | Family |  |  |  | 7.00E-04 |
| Kallotenuales | Order | N |  | - | 5.00E-04 |
| Flavobacteriaceae | Family |  |  |  | 4.00E-04 |
| Methylophilaceae | Family |  |  |  | 2.00E-04 |
| Nostocaceae | Family |  |  |  | 1.00E-04 |
| Polyangia | Class |  |  |  | 1.00E-04 |
|  |  |  |  |  |  |
|  |  | Living Mulch vs Crimson Clover | | |  |
|  |  |  | | |  |
| Verrucomicrobiaceae | Family | N |  | + | 0.0015 |
| Cryptosporangiaceae | Family |  |  |  | 1.00E-04 |
|  |  |  |  |  |  |

**Supplementary Table 8.** The association between differential family taxa of Living Mulch compared to other cover crop treatments and soil chemical data. The abundance is the relative abundance of the taxa in total community abundance.
